# A self‐gated 4D‐MRI sequence for internal target volume delineation in liver: Phantom and pre‐clinical validation

**DOI:** 10.1002/acm2.70234

**Published:** 2025-08-29

**Authors:** Shuoyan Chen, Muyu Liu, Shaobo Xue, Yiling Zeng, Bo Pang, Qi Zhang, Xuewen Hou, Hongcheng Yang, Ziyun Guan, Hong Quan, Changli Ruan, Zhiyong Yang

**Affiliations:** ^1^ Department of Medical Physics School of Physics and Technology Wuhan University Wuhan China; ^2^ Cancer Center Union Hospital Tongji Medical College Huazhong University of Science and Technology Wuhan China; ^3^ Shanghai United Imaging Healthcare Co., Ltd Shanghai China; ^4^ Department of Radiation Oncology Renmin Hospital Wuhan University Wuhan China; ^5^ Hubei Key Laboratory of Precision Radiation Oncology Wuhan China

**Keywords:** four‐dimensional computed tomography, four‐dimensional magnetic resonance imaging, liver cancer, phantom, radiotherapy, respiratory motion

## Abstract

**Background:**

The poor soft tissue resolution of four‐dimensional computed tomography (4D‐CT) limits its utility in delineating liver cancer target volumes.

**Purpose:**

To compare the consistency between four‐dimensional magnetic resonance imaging (4D‐MRI) using T1‐weighted (T1w) radial stack‐of‐stars (SOS) gradient echo (GRE) sequences and 4D‐CT in assessing tumor motion and morphology, for defining internal target volume in liver tumor radiotherapy.

**Materials and Methods:**

Position and geometric accuracy and the impact of baseline drift between 4D‐MRI (using T1w radial SOS GRE sequence) and 4D‐CT were evaluated using a motion phantom. Ten primary liver cancer patients were included for comparison. Volume, centroid position, dice similarity coefficient (DSC), and 95% Hausdorff distance (HD95) were assessed.

**Results:**

Baseline drift did not significantly affect target motion or morphology descriptions in 4D‐MRI (normal vs. drift: *p *= 0.139, DSC = 0.94 ± 0.01, HD95 = 1.82 ± 0.43) or 4D‐CT (*p *= 0.051, DSC = 0.95 ± 0.03, HD95 = 1.59 ± 0.71), but significantly impacted target volume delineation (4D‐CT: *p *= 0.040; 4D‐MRI: *p *= 0.024). Phantom studies showed consistent motion amplitude measurements between modalities for small motion patterns (2 s/10 mm; *p *= 0.192), while 4D‐MRI underestimated amplitudes in large patterns (4 s/20 mm; *p *= 0.012), consistent with patient data (small: *p *= 0.453; large: *p *= 0.004). Volume analysis revealed 4D‐MRI underestimated phantom volumes by 6.33% (2 s/10 mm) and 6.10% (4 s/20 mm) versus ground truth, contrasting with 4D‐CT's overestimations (2.01% and 2.51%). Clinically, 4D‐CT‐derived gross tumor volumes (GTVs)/internal tumor volumes (ITVs) exceeded 4D‐MRI measurements, particularly in large motion patterns (GTVs: (11.79 ± 13.13)%; ITVs: (22.08 ± 18.59)%) versus small patterns (GTVs: (9.70 ± 9.10)%; ITVs: (11.01 ± 8.05)%). Temporal stability metrics were comparable between modalities for small motion (phantom DSC *p *= 0.770, HD95 *p *= 0.131; patient DSC *p *= 0.156), with overall GTV/ITV spatial agreement (DSC: 0.79 ± 0.06 vs. 0.83 ± 0.04 for 4D‐CT vs. 4D‐MRI).

**Conclusion:**

The results show 4D‐MRI with a T1w radial SOS GRE sequence matches 4D‐CT's accuracy in target motion characterization and morphological delineation, while providing precise tumor delineation for primary liver cancer patients.

## 1 INTRODUCTION

In radiotherapy, intra‐fraction tumor displacements are primarily caused by respiration, which can induce several centimeters of motion in the cranio‐caudal (CC) direction.^1^, ^2^, ^3^ Respiratory‐induced organ and tumor displacements could lead to inaccurate target delineation, thus causing radiotherapy errors.^4^, ^5^, ^6^ Four‐dimensional computed tomography (4D‐CT) remains the gold standard for evaluating respiratory motion in radiotherapy.^7^, ^8^ It enables determination of treatment margins for tumors that move in a repeating pattern corresponding to a breathing cycle, thereby enhancing local cancer control while minimizing radiation‐induced damage to healthy tissues.^9^, ^10^, ^11^

However, 4D‐CT's limited soft‐tissue contrast often hinders clear boundary delineation between normal tissues and tumors in abdominothoracic cases, potentially leading to undetected tumors and target delineation errors. Contrast‐enhanced 4D‐CT improves lesion‐to‐tissue contrast,^12^, ^13^ though its use is restricted by contrast media (CM) allergies in some patients.^14^, ^15^ Simultaneously, contrast‐enhanced 4D‐CT performs poorly in imaging certain tumors. For colorectal liver metastases (CRLMs), 4D‐CT imaging is suboptimal due to the rapid metabolism of intravenous contrast agents.^16^ Additionally, 4D‐CT reconstruction using retrospective sorting of 3D‐CT images with external respiratory surrogates may introduce CC or anterior‐posterior (AP) motion artifacts from respiratory baseline drift.^17^, ^18^ Respiratory baseline drift refers to an involuntary shift in the average position of respiratory waveforms or organ motion trajectories over multiple respiratory cycles. This shift can lead to misalignment of anatomical structures in scanned images. Both equipment calibration deficiencies,^19^, ^20^ and unstable patient respiratory patterns,^21^ can cause respiratory baseline drift. The three‐block calibration method proposed by Liu et al.^19^ can eliminate baseline drift caused by positioning uncertainty when using a single‐block method for calibration in wall‐mounted Respiratory Gating for Scanner (RGSC) systems, controlling baseline drift within a range of 0.20 cm. The new calibration method proposed by Park et al.^20^ can reduce signal drift due to table sagging in RGSC systems with a wall mounted camera, reducing baseline drift to within 1 mm. Respiratory‐induced motion artifacts, particularly in the CC direction, could adversely affect radiotherapy outcomes.

Magnetic resonance imaging (MRI), with superior soft‐tissue contrast and sensitivity, outperforms computed tomography (CT) in delineating boundaries between organs‐at‐risk (OARs) and tumors,^22^ leading to its widespread adoption in radiation therapy. Similarly, the MRI can be divided in time to obtain four‐dimensional MRI (4D‐MRI). T1‐weighted (T1w) MRI imaging utilizes short repetition time (TR) and short echo time (TE) to highlight differences in longitudinal relaxation, enabling clear visualization of fat, vascular boundaries, and anatomical details of normal soft tissues. The shortened TR/TE parameters reduce scan duration, minimizing respiratory motion‐induced image blurring, which is a critical advantage for imaging mobile abdominal tumors where breathing artifacts degrade quality.^23^

Gradient Echo (GRE) sequences accelerate acquisition by replacing Spin Echo's 180° refocusing pulse with gradient reversals, achieving substantially shorter TE/TR. Ultra‐fast GRE variants can complete single‐slice scans in 1.7–4.1 s^24^ enabling 4D‐MRI to precisely capture respiratory‐driven organ motion (e.g., liver movement) while improving motion phase categorization accuracy.^25^ The GRE sequence is highly sensitive to inhomogeneities in the main magnetic field (B0) and gradient fields, whose sensitivity leads to geometric distortion and reduction in signal‐to‐noise ratio (SNR), ultimately degrading imaging quality.^26^ The radial stack‐of‐stars (SOS) sampling technique,^27^ addresses these limitations through golden angle‐based radial k‐space sampling. Golden‐angle sampling refers to the uniform distribution of k‐space sampling points using an irrational angular increment (111.25° or 137.51°) derived from the golden ratio (*φ* ≈ 1.618)^27^ This ensures approximately uniform k‐space coverage for any arbitrary number of consecutive radial spokes, avoiding redundant sampling. Golden‐angle radial stack‐of‐stars sampling denotes an MRI acquisition technique employing Cartesian encoding along the *z*‐axis and golden‐angle‐based radial sampling in the *xy*‐plane^28^ This angular dispersion mitigates magnetic field inhomogeneity errors and improves resistance to respiratory motion artifacts. In conclusion, 4D‐MRI using T1w radial SOS GRE sequence holds the potential to replace 4D‐CT for delineating respiratory‐induced abdominal tumor motion.^29^

Recent progress has been made in integrating 4D‐MRI into radiotherapy workflows.^30^, ^31^ Burigo and Oborn^30^ demonstrated through Monte Carlo simulations that adaptive beam delivery and plan optimization effectively correct MRI fringe field‐induced proton beam deflections in pencil beam scanning, restoring dose conformity and enabling feasible real‐time MRI guidance in proton therapy for liver and prostate cancers. However, 4D‐MRI using GRE sequences may suffer from magnetic field inhomogeneity,^32^ potentially reducing its accuracy compared to 4D‐CT in characterizing target motion and morphology. Therefore, the consistency between T1w radial SOS GRE‐based 4D‐MRI and 4D‐CT in target description requires verification for clinical use. This study compares motion characterization and morphological delineation between T1w radial SOS GRE‐based 4D‐MRI and 4D‐CT using a CT‐MRI‐compatible respiratory phantom and primary liver cancer patients, aiming to evaluate 4D‐MRI's feasibility for tumor delineation in primary liver cancer.

## 2 MATERIALS AND METHODS

### 2.1 Image acquisition

**FIGURE 1:**
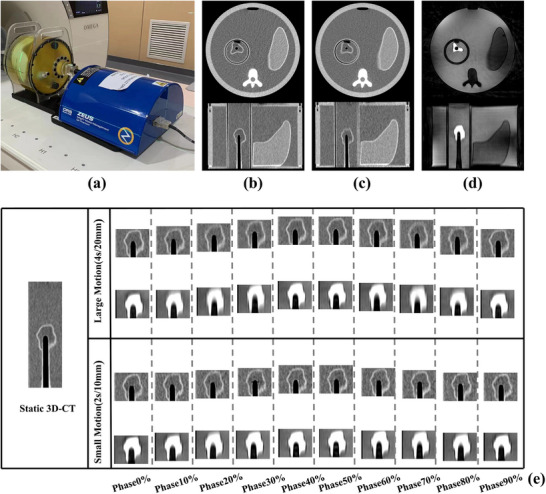
4D‐CT and 4D‐MRI images of the phantom. (a) ZEUS: MRgRT motion management QA phantom. (b) Static phantom 3D‐CT images in axial (top) and coronal (bottom) views. (c) Phase resolved phantom 4D‐CT images in axial (top) and coronal (bottom) views. (d) Phase resolved phantom 4D‐MRI images in axial (top) and coronal (bottom) views. (e) Acquired phantom target 4D‐MRI and 4D‐CT images with 10 respiratory phases under large motion pattern (4 s/20 mm) and small motion pattern (2 s/10 mm). 3D‐CT, three‐dimensional computed tomography; 4D‐CT, four‐dimensional computed tomography; 4D‐MRI, four‐dimensional magnetic resonance imaging.

The CT images were obtained using a uCT 610 sim X‐ray Computed Tomography System (Shanghai United Imaging Healthcare Co., Ltd., China). For 4D‐CT scan protocol, parameters included: 120KVp; 511mAs (60mAs) for phantom (patient); FOV: 512 × 512 mm^2^; scanning layer thickness: 2 mm (3 mm) for phantom (patient); and voxel size: 1 × 1 × 2 mm^3^ (1.172 × 1.172 × 3 mm^3^) for phantom (patient). During 4D‐CT scanning, the phantom was positioned in head‐first with an abdominal band near the motion part of the phantom to detect the respiratory phase by measuring stretching force changes. All patients were positioned head‐first and supine in customized, form‐fitting immobilization devices, and abdominal band was placed near the xyphoid process to record respiratory curves to record respiratory curves. The 4D‐CT data were sorted into 10 respiratory phase bins (*Phase*_0%_, *Phase*_10%_, …, *Phase*_90%_), where *Phase*_0%_ corresponds to end‐inspiration, *Phase*_50%_ to end‐expiration, and the remaining phases as intermediates. A static 3D‐CT phantom image was scanned as a reference (Figure 1b).

The 4D‐MRI images were acquired using a uMR Omega Magnetic Resonance Imaging System (Shanghai United Imaging Healthcare Co., Ltd., China) with a T1w radial SOS GRE sequence using diaphragmatic self‐navigation. The temporal basis was extracted from self‐navigation signals, and the spatial basis was learned from undersampled radial acquisitions using a low‐rank matrix model. A reconstructed 4D MRI image was then obtained by incorporating both spatial and temporal bases to achieve motion‐resolved imaging with high temporal resolution.^33^ Imaging parameters were: voxel size: 1 × 1 × 2 mm^3^ (1.326 × 1.326 × 3 mm^3^) for phantom (patient); scanning layer thickness: 4 mm; FOV = 304 × 304 mm^2^; flip angle = 14° (flip angle = 12°) for phantom (patient), TE = 1.77 ms (TE = 1.49 ms) for phantom (patient), TR = 3.96 ms (TR = 3.47 ms) for phantom (patient). For the same patient undergoing 4D‐MRI scanning, the identical immobilization device used in 4D‐CT scanning was employed to ensure identical patient positioning. The navigator was placed near the motion trajectory endpoint for the phantom and on the right diaphragm dome of the patient. Phase binning sorted both phantom and patient 4D‐MRI data into 10 respiratory phase bins, consistent with 4D‐CT methodology.

### 2.2 MRI‐CT respiratory motion phantom

A commercial motion phantom (ZEUS: MRgRT Motion Management QA Phantom, Sun Nuclear, USA), compatible with MRI and CT imaging, was utilized for verification (Figure 1a). The phantom body measures 25.6 cm × 32 cm × 18 cm, containing a cylindrical insert with an organic‐shaped gel‐filled target that moves along the superior‐inferior (SI) axis through background gel material.

Motion was generated using a Dynamic Motion Controller (Sun Nuclear, USA) operated by CIRS Motion Control Software (Sun Nuclear, USA). Sinusoidal signals simulated respiratory patterns, including baseline drift scenarios through linear signal modifications. Two breathing patterns were tested: small motion (2 s cycle time, 10 mm amplitude) and large motion (4 s cycle time, 20 mm amplitude). The sinusoidal motion is mathematically expressed as:

(1) $$x\left(t\right)=\frac{A}{2}\sin\left(\frac{2\pi t}{T}-\frac{\pi }{2}\right)+\frac{A}{2}$$

where *A* denotes the respiratory amplitude (*A *= 10 or 20 mm) and *T* represents the respiratory cycle (*T *= 2 or 4 s).

When incorporating a baseline drift (linear offset), the modified sinusoidal signal becomes:

(2) $$x\left(t\right)=\frac{A}{2}\sin\left(\frac{2\pi t}{T}-\frac{\pi }{2}\right)+\frac{A}{2}+at$$

where *a* is the linear drift coefficient with *a *= −0.01. To validate the impact of baseline drift, we selected a combination of 4‐s respiratory cycle (*T *= 4s) and 20‐mm respiratory amplitude (*A *= 20 mm) for experimental analysis.

Based on *T* = 4s and *A* = 20 mm in Equation (1), we varied the respiratory period (standard deviation of respiratory period = 1.5 s) to study the impact of individual heterogeneity in breathing patterns. Similarly, based on *T* = 4 s and A = 20 mm in Equation (1), we varied both the respiratory period (standard deviation of respiratory period = 1.5 s) and the respiratory amplitude (standard deviation of respiratory amplitude = 2 mm) to simulate irregular breathing and investigate its effects.

Input signals were regarded as ground truth of the target motion.

### 2.3 Patients

Ten patients (Male:Female = 6:4) with liver tumors underwent same‐day 4D‐CT and 4D‐MRI scans in the same treatment position between August 2024 and December 2024 were included. Data were retrospectively analyzed under an institutional review board‐approved protocol. Patient ages ranged from 37 to 85 years (median 62). All tumors showed clear boundaries on imaging, with single tumor per patient. 4D‐MRI scans were performed several hours after 4D‐CT. Patients with irregular breathing patterns were excluded based on respiratory curve analysis. Tumor volumes (6.24–30.50 cc from 4D‐CT) approximated the phantom's target volume (17.43 cc from static 3D‐CT).

### 2.4 Image processing

All images were imported into Treatment Planning System (TPS) version R001 (Shanghai United Imaging Healthcare Co., Ltd., China) for analysis. For phantom study, the target region of interest (ROI) was manually contoured by a single physician on static 3D‐CT image. For 4D‐CT and 4D‐MRI images, ROIs were auto‐generated and manually modified using the TPS built‐in function. Deformation fields were generated by registering the 3D‐CT (source) to each of the ten respiratory phases in 4D‐CT and 4D‐MRI (target). Contours were created by applying these deformation fields to the 3D‐CT ROI. The static 3D‐CT ROI served as the reference.

For patient study, in order to avoid tumor deformation effects, gross tumor volumes (GTVs) on 4D‐CT and 4D‐MRI were manually contoured on end‐inspiratory phase images by the same physician and auto‐generated/manually‐modified on the remaining nine phases using the TPS. Internal tumor volumes (ITVs) for 4D‐MRI were generated by combining ROIs from all ten phases. Rigid registrations were performed between corresponding phases of 4D‐CT and 4D‐MRI for comparison.

Binary ROI masks were generated using software. All phantom target and GTV contours were delineated by the same physician to ensure consistency.

### 2.5 Evaluation metrics

Patient data were dichotomized into large motion (≥1.2 cm, five patients) and small motion (<1.2 cm, five patients) on GTV centroid motion in the SI direction.

#### 2.5.1 Motion evaluation

Based on the contoured ROIs, the centroid positions of targets were measured using software. The positions of centroids were recorded to track target motion across respiratory phases. The end‐inspiration phase served as the reference, with displacement distances of other phases calculated relative to this reference.

#### 2.5.2 Morphological evaluation

The ground truth volume (*V_GT_*) of the phantom target was calculated using static 3D‐CT image. For phantom studies, absolute volume difference (*dV*) and percentage volume difference (*dV%*) between measurements and ground truth were computed using:

(3) $$dV=V_{M}-V_{GT}$$

(4) $$dV\%=\frac{V_{M}-V_{GT}}{V_{GT}}$$

where *V_M_* represents measured volumes, and *V_GT_* represents ground truth volume.

For patient study, absolute difference and the percentage difference of GTV between two modalities were calculated with:

(5) $$dV=V_{4DCT}-V_{4DMRI}$$

(6) $$dV\%=\frac{V_{4DCT}-V_{4DMRI}}{V_{4DCT}}$$

where *V_4DCT_* represents target volumes on 4DCT images, and *V_4DMRI_* represents target volumes on 4DMRI images. ITV comparisons were performed on 10 patients.

Morphological differences between ROIs were analyzed using 95% Hausdorff distance (HD95)^34^ and Dice similarity coefficient (DSC)^35^ DSC measures similarity between two binary masks:

(7) $$DSC=2*\frac{\left|A\cap \left.B\right|\right.}{\left|A\right|+\left|B\right|}$$

Where *A* and *B* denote two masks to compare.

Hausdorff distance (HD) measures maximum minimum distances between contours:

(8) $$HD=\max_{x_{i}\in \partial B}\left\{\min_{x_{j}\in \partial B}\left\{∥x_{i}-x_{j}∥\right\}\right\}$$

where $x_{i}$ is the *i*th point on contour *A* and $x_{j}$ is the point closest to $x_{i}$ on contour *B*, the 95th percentiles of the Hausdorff distance were used to reduce the impact of outliers.

For phantom studies, the static CT‐contoured ROI served as the ground truth ROI. Phase‐specific ROIs from respiratory phases were shifted to align their centroids with the ground truth ROI for HD95 and DSC calculations. These metrics were also evaluated between 4D‐CT and 4D‐MRI phase‐specific ROIs with/without baseline drift. In patient studies, GTVs contoured on end‐inspiration phase images served as references, with other nine respiratory phase GTVs shifted to match the reference centroid for HD95/DSC comparisons. Additionally, HD95 and DSC were computed between corresponding phases of 4D‐CT and 4D‐MRI data.

#### 2.5.3 Statistical analysis

SPSS 27.0 software (IBM CORP, USA) was used to do statistical analysis. The Wilcoxon signed‐rank test, Mann‐Whitney test, and *t* test were used. The reported *p* values were two‐tailed and *p* < 0.05 was considered statistically significant.

## 3 RESULTS

Figure 1e shows phantom target 4D‐MRI and 4D‐CT images with ten respiratory phases under large (4 s/20 mm) and small (2 s/10 mm) motion patterns. The static 3D‐CT image (left) provides a reference, with a ground truth volume of 17.43cc. As shown in Figure 1e, in end‐inspiratory and end‐expiratory phases, targets in both 4D‐MRI and 4D‐CT images displayed clear boundaries and minimal deformation. In other phases, targets showed varying deformation and blurred boundaries, with 4D‐MRI exhibiting more pronounced blurring and deformation than 4D‐CT. The large motion pattern caused greater distortion than the small motion pattern.

### 3.1 Phantom target motion evaluation

**FIGURE 2:**
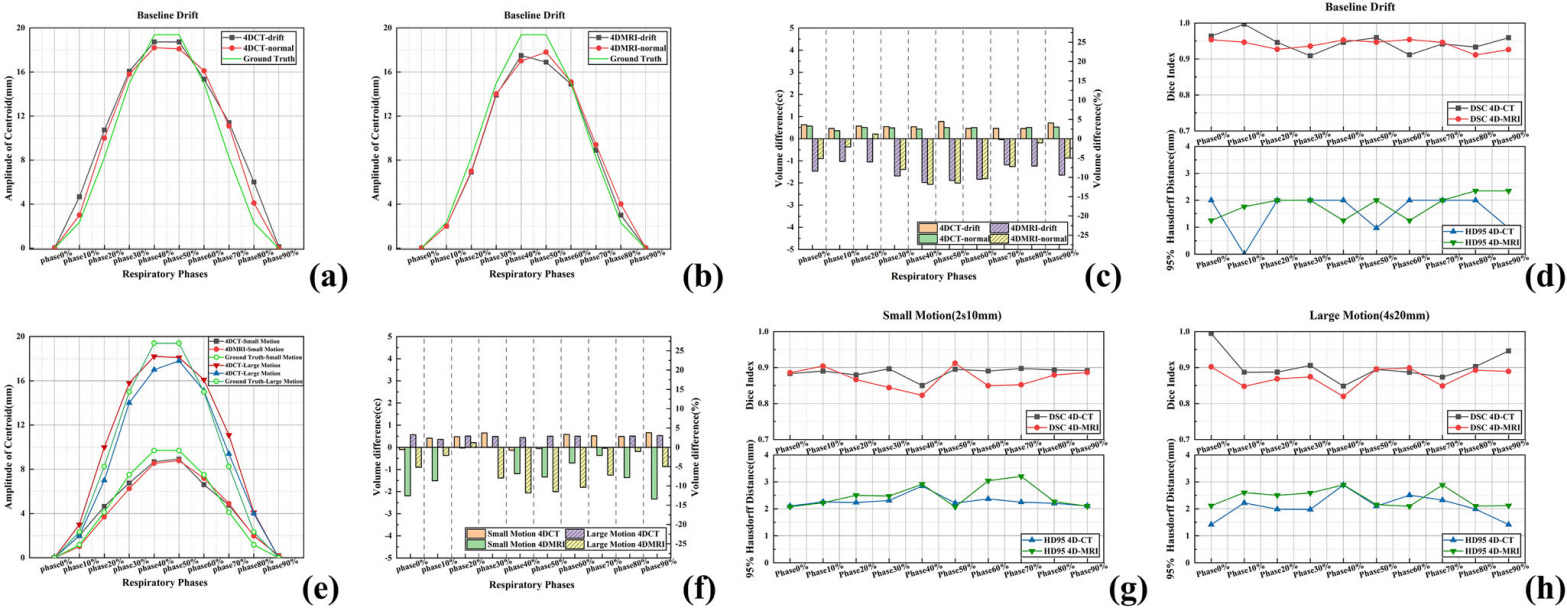
Metric differences in moving components of the phantom. (a) Normalized centroid deviation in superior‐inferior direction with and without baseline drift of 4D‐CT. (b) Normalized centroid deviation in superior‐inferior direction with and without baseline drift of 4D‐MRI. (c) The absolute difference and the percentage difference of volume between measurements and the ground truth with and without baseline drift of 4 s/20 mm respiratory pattern. (d) DSC and HD95 values calculated between ROIs with and without baseline drift for each respiratory phase. (e) Normalized centroid deviation in superior‐inferior direction of 2 s/10 mm respiratory pattern and 4 s/20 mm respiratory pattern. (f) The absolute difference and the percentage difference of volume between measurements and the ground truth of two respiratory patterns (2 and 4 s/20 mm). (g) 2 s/10 mm respiratory pattern's DSC and HD95 values calculated between ground truth and ROIs of each respiratory phase. (h) 4 s/20 mm respiratory pattern's DSC and HD95 values calculated between the ground truth and the ROIs of each respiratory phase. Ground truth volume = 17.43cc. 4D‐CT, four‐dimensional computed tomography; 4D‐MRI, four‐dimensional magnetic resonance imaging; DSC, dice similarity coefficient; HD95, the 95^th^ percentiles of the Hausdorff distance.

Figures 2a and b compare phantom target position accuracy between images with and without baseline drift. Figure 2e compares accuracy between 4D‐CT and 4D‐MRI. Table 1 summarizes target centroid motion in the SI direction for two respiratory motion patterns with/without baseline drift in both modalities. For small motion patterns, 4D‐CT and 4D‐MRI show consistent target motion descriptions in the SI direction (*p *= 0.192). Baseline drift does not affect motion descriptions in either 4D‐MRI (*p *= 0.139) or 4D‐CT (*p *= 0.051). For large motion patterns, 4D‐CT showed greater target centroid motion than 4D‐MRI in 80% of phases, with significant difference (*p *= 0.012). While no significant difference existed in deviations from true motion values between modalities for small patterns (*p *= 0.192), 4D‐MRI demonstrated greater deviation than 4D‐CT for large motion patterns (*p *= 0.012).

**TABLE 1:** Target centroid motion and volume obtained from 4D‐CT and 4D‐MRI.

	Motion^a^	Volume^b^	Motion Deviation^a^, ^c^	Volume Deviation^b^, ^c^
Baseline drift 4D‐MRI				
4D‐MRI normal	8.63/8.20/(0∼17.80)	16.37 ± 0.78	0.64/0.73/(−1.30∼2.84)	−1.06 ± 0.78
4D‐MRI drift	8.40/7.90/(0∼17.50)	15.94 ± 0.36	1.20/0.72/(−0.65∼3.70)	−1.50 ± 0.36
*p*‐value	0.107	0.024	0.051	0.024
Baseline drift 4D‐CT				
4D‐CT normal	9.64/10.55/(0∼18.20)	17.87 ± 0.18	−0.37/−0.20/(−2.40∼1.66)	0.44 ± 0.18
4D‐CT drift	10.17/11.06/(0∼18.73)	17.99 ± 0.11	−0.58/−0.20/(−2.47∼0.70)	0.56 ± 0.11
*p*‐value	0.066	0.04	0.139	0.04
Large motion				
4D‐MRI	8.63/8.20/(0∼17.50)	16.36 ± 0.78	−0.37/−0.17/(−2.40∼1.66)	−1.06 ± 0.78
4D‐CT	9.64/10.55/(0∼18.20)	17.86 ± 0.18	0.64/0.73/(−1.30∼2.84)	0.44 ± 0.18
*p*‐value	0.012	0.005	0.012	0.005
Small motion				
4D‐MRI	4.31/4.30/(0∼8.79)	16.32 ± 0.82	−0.24/−0.23/(−1.25∼0.81)	−1.10 ± 0.82
4D‐CT	4.45/4.70/(0∼8.92)	17.95 ± 0.72	−0.42/0.10/(−1.02∼0.85)	0.35 ± 0.32
*p*‐value	0.192	0.005	0.192	0.005

Abbreviations: 4D‐CT, four‐dimensional computed tomography; 4D‐MRI, four‐dimensional magnetic resonance imaging.

^a^ (mean/median/(range), mm).

^b^ (mean ± sd, cc).

^c^ Deviation from ground truth.

**FIGURE 3:**
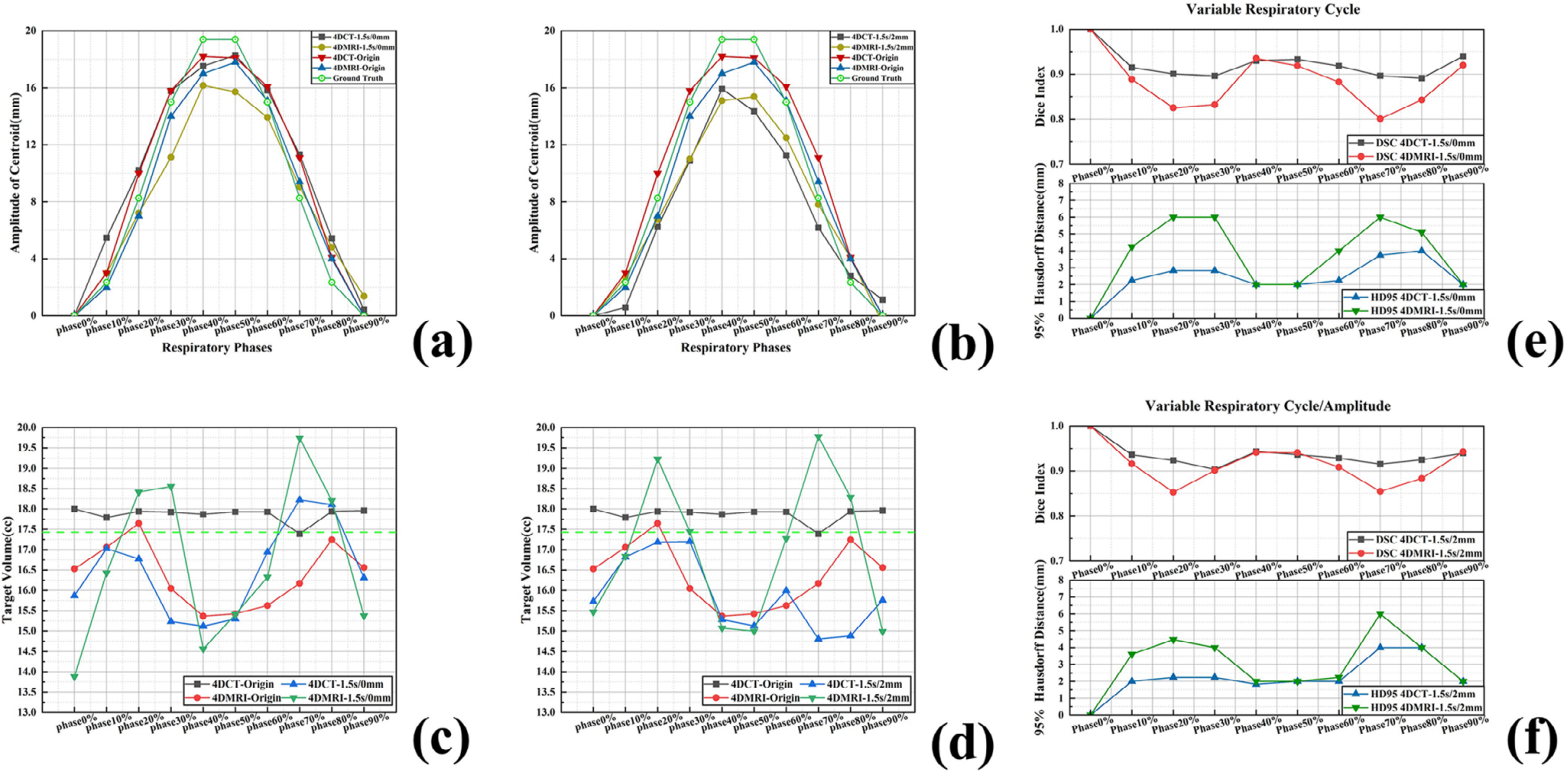
Results of phantom studies under different respiratory patterns. (a) Normalized centroid deviation of the phantom in the superior‐inferior direction under ideal sinusoidal signal and variable respiratory period signal (1.5 s/0 mm). (b) Normalized centroid deviation of the phantom in the superior‐inferior direction under ideal sinusoidal signal and variable respiratory period/amplitude signal (1.5 s/2 mm). (c) Volume of the phantom target at different phases under ideal sinusoidal signal and variable respiratory period signal (1.5 s/0 mm). (d) Volume of the phantom target at different phases under ideal sinusoidal signal and variable respiratory period/amplitude signal (1.5 s/2 mm). (e) DSC and HD95 of the phantom under variable respiratory period signal (1.5 s/0 mm). (f) DSC and HD95 of the phantom under variable respiratory period/amplitude signal (1.5 s/2 mm). DSC, dice similarity coefficient; HD95, the 95^th^ percentiles of the Hausdorff distance.

The centroid displacements of the phantom under the variable respiratory cycle signal and the variable respiratory cycle/amplitude signal compared to the ideal sinusoidal signal are shown in Figure 3a and b. For the variable respiratory cycle signal, the centroid displacement of 4D‐CT showed no statistically significant difference from that of the ideal sinusoidal signal (*p *= 0.201), while the centroid displacement of 4D‐MRI was smaller than that of the ideal sinusoidal signal. Under the variable respiratory cycle signal, the centroid displacement of 4D‐CT was larger than that of 4D‐MRI (*p *= 0.007). For the variable respiratory cycle/amplitude signal, the centroid displacements of both 4D‐CT and 4D‐MRI were smaller than that of the ideal sinusoidal signal (for 4D‐CT: *p *= 0.003; for 4D‐MRI: *p *= 0.021). Under the variable respiratory cycle/amplitude signal, no statistically significant difference was observed between the centroid displacements of 4D‐CT and 4D‐MRI (*p *= 0.171).

### 3.2 Phantom target morphological evaluation

Table 1 summaries target volumes of two respiratory motion patterns and with or without baseline drift in 4D‐CT and 4D‐MRI. For normal motion, 4D‐CT showed larger target volumes than 4D‐MRI (*p* = 0.005 for both large and small motion patterns), with average differences of 1.45cc (9.13%) for 2 s/10 mm patterns and 1.50cc (9.39%) for 4 s/20 mm patterns.

Figure 2c illustrates that the target volumes delineated by 4DMRI and 4DCT both exhibit a certain degree of overestimation or underestimation under the influence of baseline drift (*p* = 0.024 for 4D‐MRI and *p* = 0.040 for 4D‐CT). Figure 2f reveals 4D‐CT had volume deviations from ground truth volume of 0.57% (end‐inspiratory), 0.34% (end‐expiratory), and 2.81% (mid‐respiratory) for small motion, increasing to 3.27%, 2.86%, and 2.42% respectively for large motion. 4D‐MRI showed higher deviations: 12.56%, 7.68%, 5.40% (small motion) and 5.18%, 11.50%, 5.84% (large motion). 4D‐CT measurements were significantly closer to ground truth (*p *= 0.005).

Figure 2d compares DSC and HD95 between images with/without baseline drift. 4D‐CT showed DSC = 0.95 ± 0.03 and HD95 = 1.59 ± 0.71, while 4D‐MRI showed DSC = 0.94 ± 0.01 and HD95 = 1.82 ± 0.43. Both modalities maintained morphological accuracy regardless of baseline drift (*p *= 0.502 for DSC; *p *= 0.448 for HD95). Figure 2g and h shows motion pattern comparisons. For small motion, modalities showed comparable inter‐phase morphological description (*p *= 0.077 DSC; *p *= 0.131 HD95). For large motion, 4D‐CT outperformed 4D‐MRI (*p *= 0.013 DSC; *p *= 0.021 HD95).

The target volumes of the phantom across various respiratory phases under variable respiratory cycle signals and variable period/amplitude signals compared to an ideal sinusoidal signal are shown in Figure 3c and d. The DSC and HD95 for ROIs under these conditions are presented in Figure 3e and f. For variable respiratory cycle signals, the DSC of 4D‐CT was significantly greater than that of 4D‐MRI (*p *= 0.006), the DSC of 4D‐CT was significantly smaller than that of 4D‐MRI (*p *= 0.01). For variable period/amplitude signals, the DSC of 4D‐CT was significantly greater than that of 4D‐MRI (*p *= 0.039), the DSC of 4D‐CT was significantly smaller than that of 4D‐MRI (*p *= 0.027).

### 3.3 Patient motion evaluation

**FIGURE 4:**
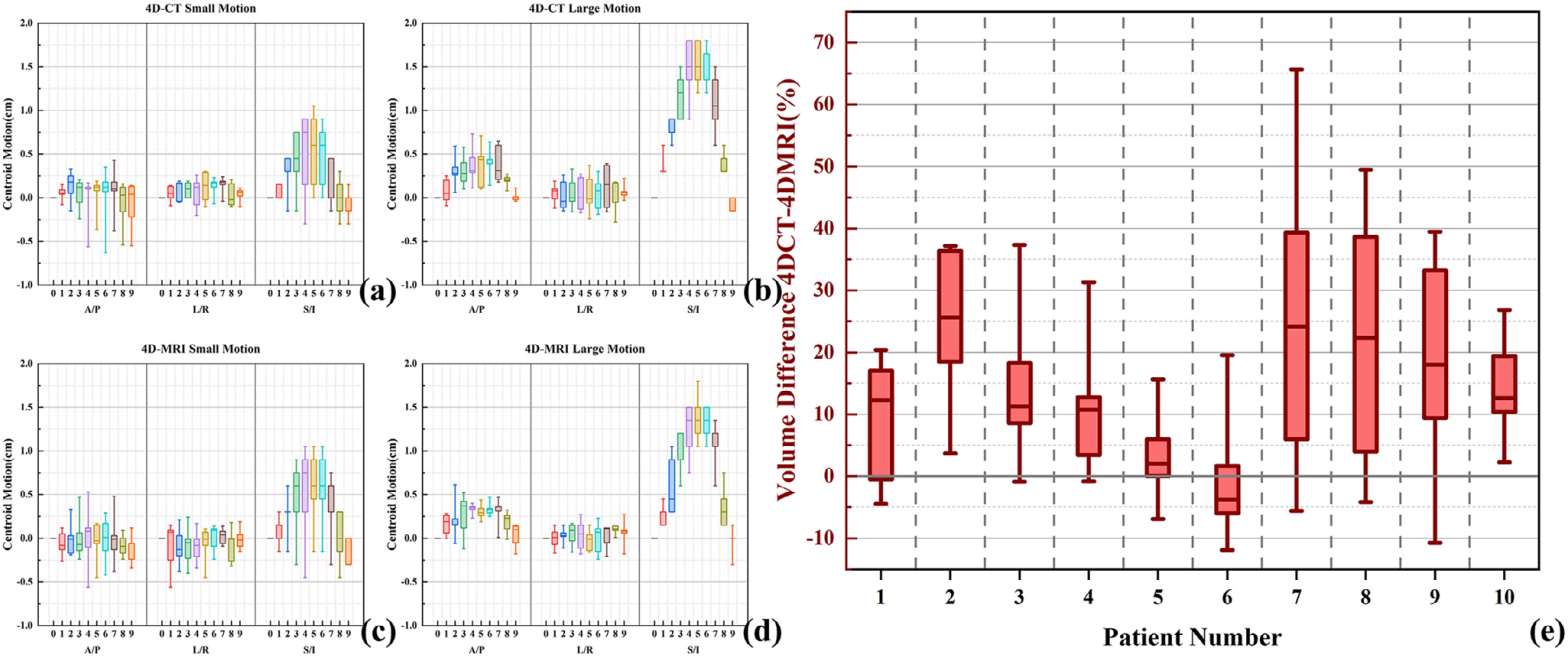
Differences in centroid and volume of patient GTV. (a) The deviation of patients’ GTV centroid on 4D‐CT images with small motion in three directions (S/I, A/P and L/R). (b) The deviation of patients’ GTV centroid on 4D‐CT images with large motion in three directions (S/I, A/P and L/R). (c) The deviation of patients’ GTV centroid on 4D‐MRI images with small motion in three directions (S/I, A/P and L/R). (d) The deviation of patients’ GTV centroid on 4D‐MRI images with large motion in three directions (S/I, A/P and L/R). (e) The average percentage difference of GTV of each patient between 4D‐CT and 4D‐MRI. 4D‐CT, four‐dimensional computed tomography; 4D‐MRI, four‐dimensional magnetic resonance imaging; S/I, Superior‐Inferior direction; A/P, Anterior‐Posterior direction; L/R, Left‐Right direction. GTV, gross tumor volume.

Figure 4a–d displays tumor centroid deviations in three directions (S/I, A/P, L/R) for two respiratory motion patterns on 4D‐CT and 4D‐MRI, with corresponding statistics in Table 2. Respiratory‐induced tumor motion primarily occurred in the SI direction. For small motion patterns, 4D‐MRI and 4D‐CT showed consistent SI direction tumor motion description (*p* = 0.453). For large motion patterns, 80% of phases (40/50) demonstrated greater or comparable SI direction motion in 4D‐CT versus 4D‐MRI, with significant inter‐modality differences (*p* = 0.004).

**TABLE 2:** Patients’ GTV, ITV, and GTV centroid motion obtained from 4D‐CT and 4D‐MRI.

	4D‐CT	4D‐MRI		*p*‐value
GTV centroid motion^a^				
Large motion				
S/I direction	0.82/0.90/(−0.15∼1.80)	0.71/0.75/(−0.30∼1.80)		0.004
A/P direction	0.24/0.21/(−0.09∼0.73)	0.21/0.24/(−0.18∼0.61)		0.229
L/R direction	0.05/0.00/(−0.28∼0.39)	0.03/0.05/(−0.24∼0.27)		0.200
Small Motion				
S/I direction	0.25/0.15/(−0.30∼1.05)	0.27/0.30/(−0.45∼1.05)		0.453
A/P direction	0.02/0.09/(−0.20∼0.30)	−0.03/−0.01/(−0.56∼0.53)		0.035
L/R direction	0.07/0.09/(−0.56∼0.24)	−0.05/−0.01/(−0.56∼0.24)		<0.001
Volume^b^				
Large motion			Deviation^c^	
GTV	16.40 ± 8.13	14.10 ± 6.75	11.79 ± 13.13	<0.001
ITV	31.03 ± 15.33	25.36 ± 11.78	22.08 ± 18.59	0.037
Small motion				
GTV	17.89 ± 10.52	15.10 ± 9.55	9.70 ± 9.10	<0.001
ITV	25.43 ± 13.89	23.49 ± 14.35	11.01 ± 8.05	0.001

Abbreviations: 4D‐CT, four‐dimensional computed tomography; 4D‐MRI, four‐dimensional; GTV, gross tumor volume; ITV, internal tumor volume.

^a^ (mean/median/(range), cm).

^b^ (mean ± sd, cc).

^c^ (mean ± sd,%).

### 3.4 Patient morphological evaluation

**FIGURE 5:**
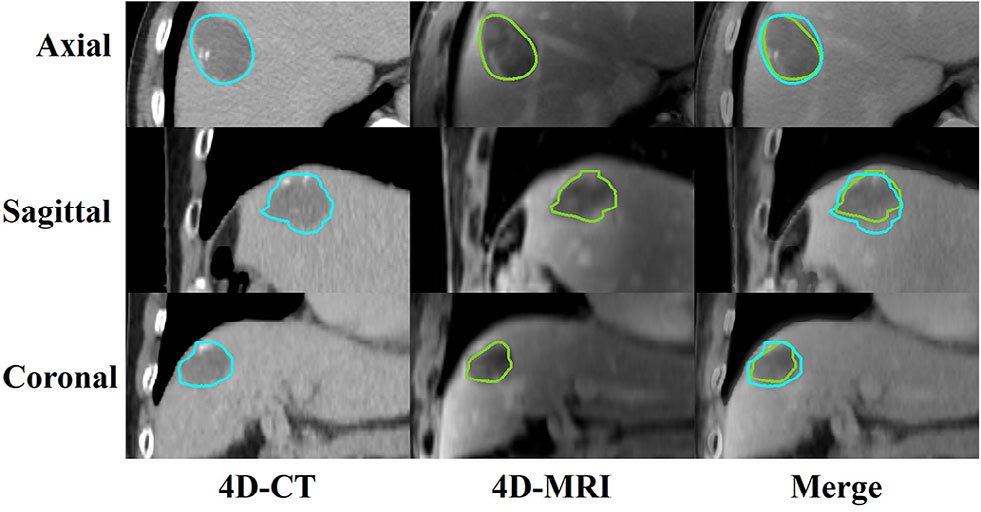
Images of GTV in liver cancer patients. Multiplanar (axial, sagittal, and coronal planes) four‐dimensional computed tomography (4D‐CT), four‐dimensional magnetic resonance imaging (4D‐MRI), and fused imaging datasets acquired at the end‐expiration phase from a selected patient case.

Figure 4e shows GTV volume percentage differences between modalities. Table 2 summarizes GTV and ITV comparisons. For small motion patterns, 4D‐MRI showed smaller GTV (1.72cc/9.70%, *p *< 0.001) and ITV (1.94cc/11.01%, *p *= 0.001) than 4D‐CT. Similar trends occurred for large motion patterns (GTV: 1.94cc/11.79%, *p *< 0.001; ITV: 5.68cc/22.08%, *p *= 0.027). Figure 5 illustrates tumor volume delineation discrepancies.

**FIGURE 6:**
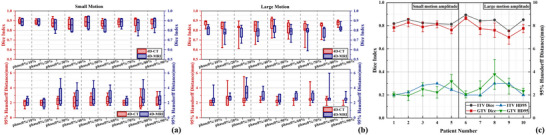
Dice similarity coefficient and Hausdorff distance for patient GTV and ITV. (a) to (b) are two respiratory patterns’ DSC and HD95 values calculated between end of inspiratory phase's ROI and other nine respiratory phases’ ROI on 4D‐CT and 4D‐MRI. Phase0%/10% represents the comparison between 0% phase and 10% phase, and so on. (a) Small motion DSC and HD95. (b) Large motion DSC and HD95. (c) ITV and GTV's DSC and HD95 values calculated between 4D‐CT and 4D‐MRI. DSC, dice similarity coefficient; HD95, the 95^th^ percentiles of the Hausdorff distance. 4D‐CT, four‐dimensional computed tomography; 4D‐MRI, four‐dimensional magnetic resonance imaging; ITV, internal tumor volume; GTV, gross tumor volume.

Figure 6a and b shows large and small motion patterns’ DSC and HD95 values calculated between end of inspiratory phase's ROI and other respiratory phase's ROIs on two modalities, and Figure 6c shows ITV and GTV's DSC and HD95 values calculated between 4D‐CT and 4D‐MRI. For small motion pattern, average phase resolved DSC is 0.88 ± 0.04 in 4D‐CT, and 0.87 ± 0.06 in 4D‐MRI, the difference is not statistically significant (*p* = 0.156). For large motion pattern, average phase resolved DSC is greater in 4D‐CT (0.83 ± 0.08) than that in 4D‐MRI (0.79 ± 0.07), which indicates that 4D‐CT can more consistently describe target morphology between phases than 4D‐MRI (*p* < 0.001). And for two motion patterns, HD95 in 4D‐MRI (2.93 ± 1.33 mm for small motion and 2.89 ± 0.88 mm for large motion) is greater than that in 4D‐CT (2.64 ± 1.26 mm for small motion and 2.38 ± 0.84 mm for large motion), which is statistically significant (*p* = 0.013 for small motion and *p* = 0.002 for large motion). Figure 6c shows no significant ITV delineation differences between motion patterns (DSC:0.83 ± 0.02 vs. 0.84 ± 0.05, *p *= 0.421; HD95:2.50 ± 0.41 mm vs. 2.40 ± 0.54 mm, *p *= 0.690).

## 4 DISCUSSION

In this study, motion phantom and ten patients with liver tumors were studied to prove that 4D‐MRI using T1w radial SOS GRE sequence and 4D‐CT have certain consistency in describing target motion and target morphology. Therefore, the non‐contrast T1w‐4D‐MRI with radial SOS GRE sequence demonstrated comparable accuracy in respiratory motion measurement, and high accuracy in tissue properties quantification. It shows potential to be used for internal target volume delineation in radiotherapy for liver tumors. Furthermore, the 4DMR technique leverages a partial separable low‐rank approach, which places minimal demands on system performance. Unlike some advanced imaging methods, it does not require exceptional B0 homogeneity. This makes the technique versatile across different magnetic field strengths.

Findings of our research demonstrate that while both 4D‐MRI using a T1w radial SOS GRE sequence and 4D‐CT accurately characterize target motion unaffected by baseline drift, both modalities exhibit sensitivity to baseline drift in target volume delineation. 4D‐CT relies on external respiratory surrogates for respiratory signal acquisition. Baseline drift may disrupt the correspondence between external surrogates and true internal respiratory motion, potentially reducing imaging quality and introducing volume estimation errors.^36^ In contrast, 4D‐MRI employs diaphragmatic self‐navigation to derive respiratory curves, theoretically mitigating baseline drift effects.^36^, ^37^ However, magnetic field inhomogeneity‐induced phase shifts in internal navigator signals can lead to respiratory phase binning misalignment. For example, diaphragm‐region signal fluctuations may cause erroneous image sequence sorting and motion artifacts,^38^ thereby compromising volume delineation accuracy. DSC and HD95 analyses confirm comparable robustness to baseline drift between the two modalities.

The study results indicate that 4D‐CT is less affected by irregular respiratory cycles in describing motion, whereas 4D‐MRI is more significantly impacted. A possible reason is that under irregular breathing, 4D‐MRI's uniform respiratory binning results in varying amounts of k‐space data across phases. Phases with insufficient data may exhibit misalignment of anatomical structures after reconstruction, compromising image quality. In contrast, 4D‐CT does not rely on k‐space data for reconstruction and is thus less susceptible to this issue. Both 4D‐CT and 4D‐MRI are influenced by irregular respiratory cycles/amplitudes in describing target motion and target volume. For 4D‐CT, since both phantom motion and treatment couch motion occur in the SI direction, irregular respiratory amplitudes cause anatomical overlaps or gaps, leading to underestimation or overestimation of volumes.^39^ This results in target volume fluctuations, as shown in Figure 3d. For 4D‐MRI, irregular amplitudes cause motion artifacts due to erroneous image sorting. Larger motion artifacts occur in mid‐inhalation phases with greater target motion, while smaller artifacts appear in end‐exhalation/inhalation phases with minimal motion, similarly causing volume fluctuations. This results in larger deviations in target delineation for mid‐respiratory phase images, whereas smaller deviations for end‐inspiration/end‐expiration phase delineations.

In phantom study, respiratory motion amplitude was shown to influence consistency between 4D‐CT and 4D‐MRI in motion characterization. Both modalities demonstrated concordance for small motion patterns, but 4D‐CT measured greater target centroid displacement than 4D‐MRI in large motion patterns, as shown in Figure 2e and Table 1. Patient studies revealed similar trends: 4D‐MRI and 4D‐CT showed comparable tumor motion measurements in the SI direction for small displacements, while 4D‐MRI recorded smaller amplitudes than 4D‐CT in large motion patterns, as shown in Figure 4a–d and Table 2. And for small motion patterns, there was no significant difference in the deviations of 4D‐MRI and 4D‐CT from the true value of target motion, whereas for large motion patterns, 4D‐MRI exhibited greater deviation from the true value of target motion compared to 4D‐CT, as shown in Table 2. A key technical difference emerged in slice thickness implementation. Though both modalities maintained equivalent reconstructed slice thicknesses (2 mm phantom/3 mm patient), 4D‐MRI's actual scanning slice thickness was 4 mm prior to reconstruction. This thicker acquisition likely exacerbated partial volume effects (PVEs) in 4D‐MRI, particularly during large motions where PVE‐induced motion underestimation becomes more pronounced. In contrast, 4D‐CT's inherently thinner slices minimized PVEs. Quantitative analysis showed clinically acceptable discrepancies. Phantom experiments two modalities revealed −0.004 mm mean centroid motion difference from ground truth (range: −2.97 to 2.83 mm), with only 5% phases (two out of forty phases) exceeding 2 mm deviation. Patient data demonstrated that 84% of respiratory phase discrepancies between modalities in large motion patterns remained below the 3 mm reconstructed slice thickness. Given both modalities’ identical voxel dimensions (1 × 1 × 2 mm^3^), these variations fall within expected spatial resolution limits. These findings suggest that while 4D‐MRI may modestly underestimate large motions compared to 4D‐CT, the absolute differences remain sub‐voxel in scale. Therefore, 4D‐MRI and 4D‐CT can be considered clinically equivalent for target motion characterization. The phantom studies by Wang et al.^40^ showed similar results.

Phantom study results demonstrated that 4D‐MRI using T1w radial SOS GRE sequences systematically underestimated target volumes under both large and small respiratory motion patterns, while 4D‐CT maintained relatively good accuracy in delineating ground truth volumes, as shown in Figure 2f and Table 1. For the underestimation of target volume by 4D‐MRI, a possible explanation is that in the phantom study, the sampling resolution of 4D‐MRI was 1 × 1 × 4 mm^3^, and although it was reconstructed to 1 × 1 × 2 mm^3^ resolution, the lower spatial resolution may lead to insufficient imaging accuracy for small structures or boundary regions, resulting in smaller volume measurements. As for the overestimation of target volume by 4D‐CT, a possible explanation is that the use of an external respiratory surrogate may cause errors in the phase binning process of 4D‐CT, causing the reconstructions to also reflect the tumor positions from the other phases. Both modalities showed phase‐dependent performance: 4D‐CT achieved superior accuracy during end‐inspiration and end‐expiration phases compared to mid‐respiratory phases, consistent with prior findings.^41^ This likely occurs due to slower respiratory motion at extreme phases, reducing motion artifacts and improving delineation accuracy. In contrast, 4D‐MRI tends to systematically underestimate target volumes during end‐expiratory and end‐inspiratory phases but shows better agreement with ground truth volume in mid‐respiratory phases. This discrepancy is not necessarily indicative of superior accuracy in mid‐phases for 4D‐MRI. Instead, it likely stems from two competing factors. The larger scan slice thickness in 4D‐MRI exacerbates PVEs and spatial misregistration along the phase encoding direction caused by reduced temporal synchronization between gradient field switching and target motion, causing systematic volume underestimation. Meanwhile, motion artifacts during high‐velocity phases induce volume overestimation. These opposing errors offset mid‐phase underestimation, creating an illusion of improved accuracy. Furthermore, 4D‐MRI's thicker slices increased susceptibility to motion artifacts, resulting in greater inter‐phase volumetric variability than 4D‐CT.

The results of patient study demonstrated that 4D‐CT delineates larger GTV and ITV compared to 4D‐MRI across both large and small motion amplitude patterns, a trend consistent with phantom‐based validation study outcomes, as shown in Figure 4e and Table 2. It is noteworthy that several outliers exist in Figure 4e, particularly for patient seven, where the percentage volume deviation even exceeds 60%. Since the GTV sizes of the selected patients range from 6.24 cc to 30.50 cc, for patients with smaller GTVs (e.g., 6.24 cc), an absolute volume deviation of 3–4 cc can result in a percentage volume deviation of 50%–60%. Studies by other researchers have demonstrated concordant findings.^42^, ^43^ In a cohort of 23 patients with primary liver cancer, Chen et al.^42^ revealed that 4D‐CT overestimates GTV and ITV compared to 4D‐MRI. Similarly, comparative study between CT‐derived GTV volumes and pathological specimen measurements showed a volumetric overestimation in CT‐based tumor delineation to actual surgical specimens.^43^ Notably, the discrepancies in tumor volume delineation between 4D‐CT and 4D‐MRI observed in clinical patient data demonstrate more pronounced variations compared to those documented in phantom measurements. A hypothetical explanation is that the soft tissue contrast of CT images is relatively low, particularly in areas of necrosis or hemorrhage, where the density difference between tumors and surrounding liver tissue may be insignificant, leading to blurred boundaries.^44^ During contour delineation, surrounding edema, inflammatory regions, or ambiguous areas might be misjudged as tumor tissue, resulting in volume overestimation. In contrast, T1‐weighted MRI demonstrates superior soft tissue resolution. It clearly revealing the tumor's pseudocapsule and internal structures (such as fat and necrosis), thereby enabling more precise definition of tumor boundaries. Combined with the phantom study findings, we recommend using larger margin when using ITV based on 4D‐MRI images. For the phantom target volume (17.43 cc) used in this study, a larger expansion of the ROI margin across all 4D‐MRI phases approximates the actual phantom target volume. However, the extent of clinical margin expansion may depend on GTV size and requires further investigation.

Volumetric measurements alone inadequately captured shape deformations between respiratory phases (identical volumes with different shapes), necessitating complementary evaluation using DSC and HD95. Analysis of phantom and patient datasets revealed that DSC and HD95 variations paralleled volumetric changes across both small and large motion patterns: end‐inspiratory and end‐expiratory phases demonstrated superior target morphology delineation compared to intermediate phases, as shown in Figure 2g and f. This trend was more pronounced in large motion patterns, indicating greater susceptibility of 4D‐MRI's inter‐phase morphological consistency to motion magnitude variations, which aligns with respiratory phantom‐based analyses by Wang et al.^40^ In patient evaluations, DSC and HD95 comparisons between ITV and GTV in 4D‐CT versus 4D‐MRI showed no significant differences across respiratory motion patterns. According to the research findings of Chen et al.^45^ regarding the relationship between DSC and dose differences, the GTV/ITV structural differences between 4D‐CT/4D‐MRI reflected by DSC obtained in this study were considered clinically acceptable. Although 4D‐MRI using T1w radial SOS GRE sequence is slightly inferior to 4D‐CT in depicting target motion and morphology, analysis of DSC and HD95 metrics between GTV/ITV contours demonstrates its clinical comparability. 4D‐MRI achieves comparable accuracy to 4D‐CT for hepatic tumor delineation, offering a viable alternative for liver cancer contouring in clinical practice.

This study has several limitations. 4D‐MRI slice thickness was limited to 4 mm due to scanner/sequence constraints, with thicker slices causing partial volume effects that reduced analytical accuracy. Subsequently, we will focus on eliminating the limitation of 4 mm scan slice thickness, such as achieving 2 mm or thinner slices through sequence parameter optimization. Additionally, the impact of 4 mm slice thickness can be mitigated by exploring post‐processing techniques. The patent by Stazzone et al.^46^ proposes a rapid interleave overlap technique (RIOT), which synthesizes 2 mm slice thickness images by overlapping 50% of the original 4 mm slices, significantly enhancing effective resolution. Furthermore, deep learning methods can be employed to improve inter‐slice resolution.^47^ Although patient GTVs were aligned with the phantom's target volume, the phantom's single motion target type restricted comparative evaluations of 4D‐CT and 4D‐MRI performance in analyzing motion characteristics and morphological delineation across diverse targets. The phantom's motion mechanism only permitted one‐dimensional reciprocating movement, lacking simulation capability for AP or lateral motions, or complex tumor behaviors like tissue deformation or rotation. The potential dosimetric benefits of 4D‐MRI for target delineation also remain unexamined. These limitations require further exploration to fully evaluate 4D‐MRI's clinical utility.

## 5 CONCLUSION

This study demonstrated that 4D‐MRI using T1w radial SOS GRE sequence can achieve comparable accuracy to 4D‐CT in both target motion characterization and morphological delineation. Attributable to its exceptional soft‐tissue contrast resolution, 4D‐MRI demonstrates the potential to supersede 4D‐CT for delineating abdominal malignancies impacted by respiratory motion.

## AUTHOR CONTRIBUTIONS

**Shuoyan Chen**: Writing—review & editing; writing—original draft; visualization; validation; investigation; formal analysis. **Muyu Liu**: Visualization; investigation; methodology; conceptualization. **Shaobo Xue**: Software; formal analysis. **Yiling Zeng**: Writing—review & editing; software; investigation. **Bo Pang**: Writing—review & editing. **Qi Zhang**: Writing—review & editing. **Xuewen Hou**: Writing—review & editing; hardware support. **Hongcheng Yang**: Writing—review & editing; hardware support. **Ziyun Guan**: Hardware support. **Hong Quan**: Writing—review & editing; supervision; investigation. **Changli Ruan**: Writing—review & editing; supervision. **Zhiyong Yang**: Writing review & editing; supervision; project administration; methodology.

## CONFLICT OF INTEREST STATEMENT

The authors declared no conflict of interest.

## ETHICAL STATEMENT

All patients included in this study were enrolled in a retrospective data collection protocol [2023]IEC(254) approved by the institutional review board of Union Hospital, Tongji Medical College, Huazhong University of Science and Technology.
